# Extracellular Vesicles: From Biology to Biomedical Applications

**DOI:** 10.3390/bioengineering6030079

**Published:** 2019-09-06

**Authors:** Esther Serrano-Pertierra, Maria Carmen Blanco-López

**Affiliations:** Department of Physical and Analytical Chemistry, University of Oviedo, 33006 Oviedo, Spain

Legend says that Philippides ran to Athens to announce the victory against the Persians in the Battle of Marathon. The Battle of Karansebes was a misunderstanding that ended up with the loss of up to 1200 Austro-Hungarian soldiers towards the end of the 18th century. Almost 200 years later, the SS Californian could have rescued hundreds of Titanic passengers.

For better or worse, communication (or lack thereof) is at the center of these stories as it is for every aspect of life, starting from both prokaryotes and eukaryotes. Cells must communicate and process information to correctly respond to their microenvironment. 

The capacity of extracellular vesicles (EVs) to mediate intercellular communication has drawn attention from some of the first studies. These membranous structures are released by cells, containing different types of molecules (proteins, nucleic acids, lipids, and sugars), which can be transferred to other cells, thereby affecting or influencing the target cell function.

Research studies have highlighted their role in both physiological and pathological processes. Therefore, EVs have emerged as potential biomarkers in several diseases. In this sense, understanding the biology of EVs is crucial to elucidate how this communication process works and the outcome of the message transmitted (while keeping the safety of the messenger). 

Investigation into EVs is as challenging as EV isolation, characterization, and functional analysis. This special issue collects representative work in the field. Starting from isolation, some of the current available methods provide different results with the same samples, and therefore downstream studies could be affected by this step. This highlights the need for simple and standardized protocols for this crucial step.

Quantification and multiple marker characterization are required in order to investigate their potential role as biomarkers. The common methods used for this purpose are discussed in a very complete review describing the principles of the most used techniques and outlining the emerging technologies. 

The rest of the papers in this Special Issue (Table 1) show different examples of the involvement of EVs in different biological processes and their potential biomedical applications: EVs from oviduct secretions, as modulators of gamete/embryo interactions, could improve infertility problems at assisted reproductive therapy; stem cell EVs show promising results to restore epithelial tissues, improve wounds scarring, and smooth signs of skin aging; and finally, a review article on EVs from protozoa discusses their potential uses for vaccination and drug delivery. 

In future EV therapies, communication is the key for success.

We would like to thank all the authors who have contributed, the reviewers, the editorial office, and editors that assisted this Special Issue.

## Figures and Tables

**Table 1:** Publications in special issue.

Topic	Reference
Stem Cell Extracellular Vesicles in Skin Repair	Ferreira and Gomes [1]
Extracellular Vesicle Quantification and Characterization: Common Methods and Emerging Approaches	Hartjes et al. [2]
Characterization of Plasma-Derived Extracellular Vesicles Isolated by Different Methods: A Comparison Study	Serrano-Pertierra et al. [3]
Extracellular Vesicles from the Protozoa *Acanthamoeba castellanii*: Their Role in Pathogenesis, Environmental Adaptation and Potential Applications	Gonçalves et al. [4]
Extracellular Vesicles in the Oviduct: Progress, Challenges and Implications for Reproductive Success	Almiñana and Bauersachs [5]
